# Mapping heterogeneous polarity in multicompartment nanoparticles

**DOI:** 10.1038/s41598-018-35257-y

**Published:** 2018-11-20

**Authors:** Francesco Palomba, Damiano Genovese, Luca Petrizza, Enrico Rampazzo, Nelsi Zaccheroni, Luca Prodi

**Affiliations:** Dipartimento di Chimica “Giacomo Ciamician”, Università di Bologna, Via Francesco Selmi, 2, 40126 Bologna, Italy

## Abstract

Understanding polarity gradients inside nanomaterials is essential to capture their potential as nanoreactors, catalysts or in drug delivery applications. We propose here a method to obtain detailed, quantitative information on heterogeneous polarity in multicompartment nanostructures. The method is based on a 2-steps procedure, (i) deconvolution of complex emission spectra of two solvatochromic probes followed by (ii) spectrally resolved analysis of FRET between the same solvatochromic dyes. While the first step yields a list of polarities probed in the nanomaterial suspension, the second step correlates the polarities in space. Colocalization of polarities falling within few nanometer radius is obtained via FRET, a process called here nanopolarity mapping. Here, Prodan and Nile Red are tested to map the polarity of a water-dispersable, multicompartment nanostructure, named PluS nanoparticle (NPs). PluS NPs are uniform core-shell nanoparticles with silica cores (diameter ~10 nm) and Pluronic F127 shell (thickness ~7 nm). The probes report on a wide range of nanopolarities among which the dyes efficiently exchange energy via FRET, demonstrating the coexistence of a rich variety of environments within nanometer distance. Their use as a FRET couple highlights the proximity of strongly hydrophobic sites and hydrated layers, and quantitatively accounts for the emission component related to external water, which remains unaffected by FRET processes. This method is general and applicable to map nanopolarity in a large variety of nanomaterials.

## Introduction

Polarity and proticity of confined nanoenvironments play a fundamental role in many chemical and biochemical processes, since they govern the strength of noncovalent interactions such as H-bonds, electrostatic and van der Waals interactions or π-π stacking. Processes driven by local environmental heterogeneity, are of different nature and of great importance such as enzymatic catalysis^1–4^, molecular recognition, self-assembly of polymers, micelles and vesicles^5^, molecular transport and delivery^6^, and bottom-up formation of nanostructures and nanomaterials^7–10^. Nanomaterials are expressing a rich potential inasmuch as they can implement such heterogeneity in polarity at the nanoscale, featuring several environments with different physicochemical properties in a single structure. This richness is comparable to the complexity of biological species such as proteins and DNA, and it is at the basis of their peculiar versatility. They are able, in fact, to perform complex functions, such as drug delivery and targeted transport^6,11–13^, adsorption of pollutants^14^, adhesion and colloidal stabilization^15,16^. Despite the extensive production of novel nanomaterials composed of several compartments with different characteristics^13,17^, the characterization of their polarity distribution at the nanoscale is so demanding to remain often an assumption based on their chemical structure and morphology. Fluorescent solvatochromic dyes have been used in seminal works to investigate polarity of micelles and vesicles^18,19^. Similar information could also be sought for more elaborate nanomaterials composed of multiple domains. Nonetheless, the complexity of such architectures makes it difficult to obtain a correct description of the multiple nanoenvironments, and to distinguish which environment is actually being probed by the dye. The current models used to describe micropolarity, including those based on the Kamlet-Taft parameters (hydrogen bond donor α, hydrogen bond acceptor β, and polarizability π*), provide only an average description of polarity^20–29^. Some efforts have also been done to isolate the contribution of the bulk solvent^30^. A correct description of complex multicompartment nanostructures, on the contrary, requires to find unambiguous interpretation of multi-component data. In this contribution, we describe a method based on fluorescence spectra deconvolution and FRET analysis that yields polarity mapping of complex nanoarchitectures.

Our approach overcomes the limits of current methods based on Kamlet-Taft parameters, since it allows to simultaneously quantifying a wide range of polarities featured by the nanomaterial. In addition, the method provides correlation in space of polarity and proticity information, identifying those compartments -located few nanometers apart- that feature different polarity and exchange excitation energy. In a first step, we performed spectral deconvolution of the emission spectra of solvatochromic fluorescent probes: the complex spectrum of each solvatochromic probe in a multicompartment nanostructure is fitted using a set of spectra of the same probe in pure solvents. The coefficients obtained by the fitting, quantify the fraction of dyes probing, inside the nanostructure, a polarity similar to that of each pure solvent. The number and type of solvents used for the fitting set determine the resolution of the technique and should be adequately chosen according to the polarity gradient to be explored and quantified. The second step of our proposed method allows to estimate the approximate distance between probed compartments. This is possible carefully selecting at least two solvatochromic fluorescent dyes with suitable photophysical features to give efficient FRET processes in all combinations of environments. The multiple FRET contributions are sorted by polarity via spectral deconvolution. As a result, the estimation of the energy transfer efficiency for each individual spectral component of the probes yields a fine mapping of the nanoenvironment polarities within the nanostructure.

## Results and Discussion

### Choice of nanostructure and of solvatochromic FRET couple

**Figure 1:**
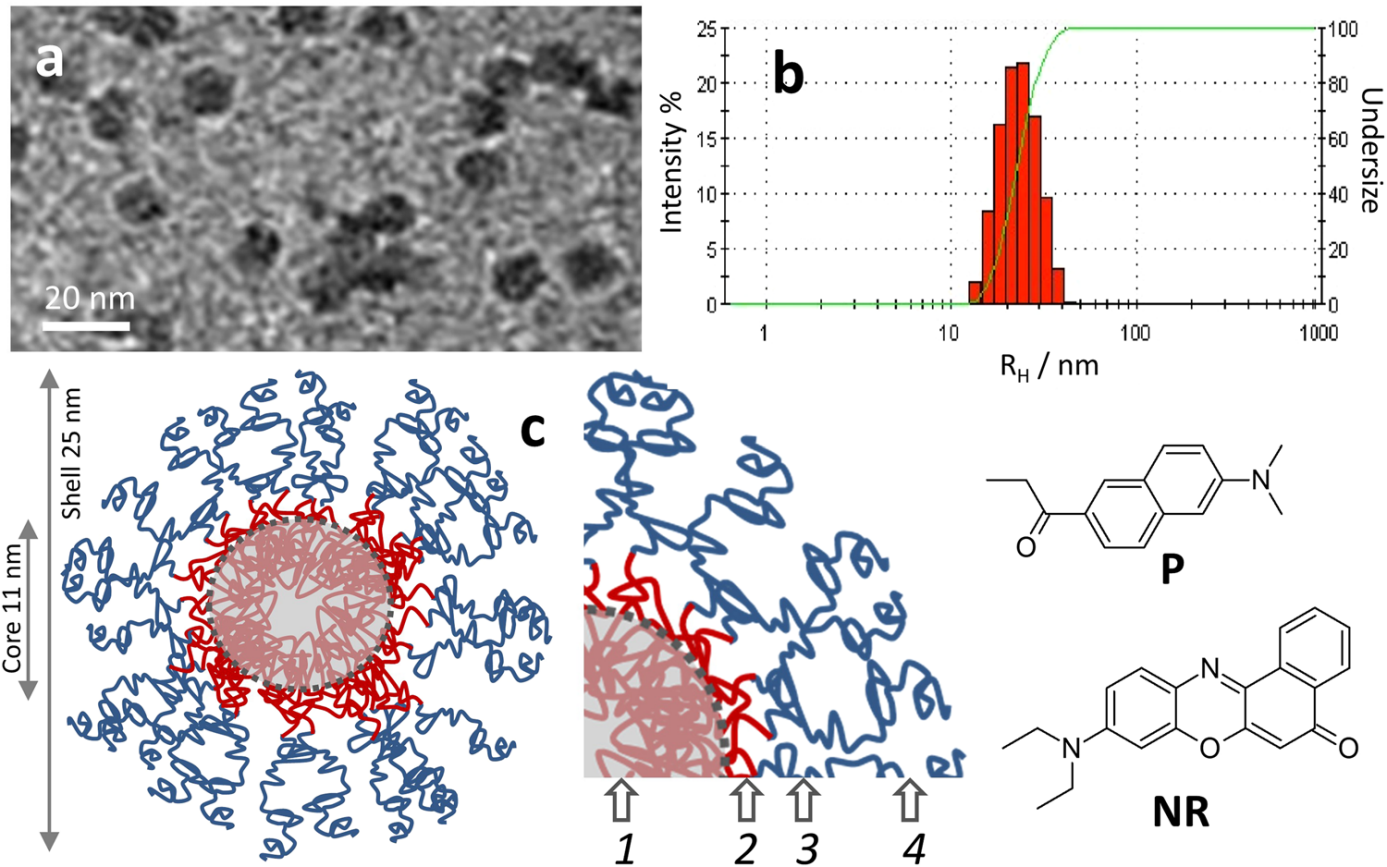
TEM micrograph (**a**) and hydrodynamic radius distribution obtained by DLS (**b**) of PluS NPs. Cartoon of multicompartment nanostructures PluS NPs (**b**) probed by the luminescent solvatochromic dyes Prodan (**P**) and Nile Red (**NR**). Numbers 1–4 indicate the compartments of PluS NPs, specifically: (1) dense silica core embedding PPO chains; (2) PPO brushes emerging from the silica core; (3) interfacial region with PPO and PEG; (4) external layer with PEG brushes.

Our aim, because of the high interest in the design and characterization of new functional materials, was to find a method to map heterogeneous polarity in multicompartment nanostructures. We tested this method by investigating a multilayer nano-architecture, previously named “PluS NPs”, composed of a silica core grown into a micelle of the triblock copolymer Pluronic F127^31–33^. In earlier works we proved that this nanomaterial is composed of a dense silica core and of a soft polymeric shell. This last one, among other things, is responsible for the long-term colloidal stability in water, and for the ability of these nanoparticles to host hydrophobic moieties close to the internal silica core^34–36^. The shell should hence display both hydrophobic hosting sites and a hydrophilic surface ensuring favorable interaction with external water (Figs 1 and SI1). Despite the consolidated knowledge on the structure, behaviors and abilities of this nanoarchitecture, the intimate distribution of its environments with different polarity and proticity still remains to be fully elucidated. Previous data on morphology and on chemical properties of the components suggest a possible model for the nanoarchitecture that consists of a dense silica shell, in which the hydrophobic polypropylene oxide (PPO) segments are deeply buried, and of a permeable soft polymeric shell^31,34,36^. The shell itself might be composed of two sections: a hydrophobic inner layer close to the silica, made of PPO (the part that is not completely buried in the silica network) and an outer layer made of the PEG terminations, which could be partly hydrated owing to its good affinity with water (Fig. 1).

**Figure 2:**
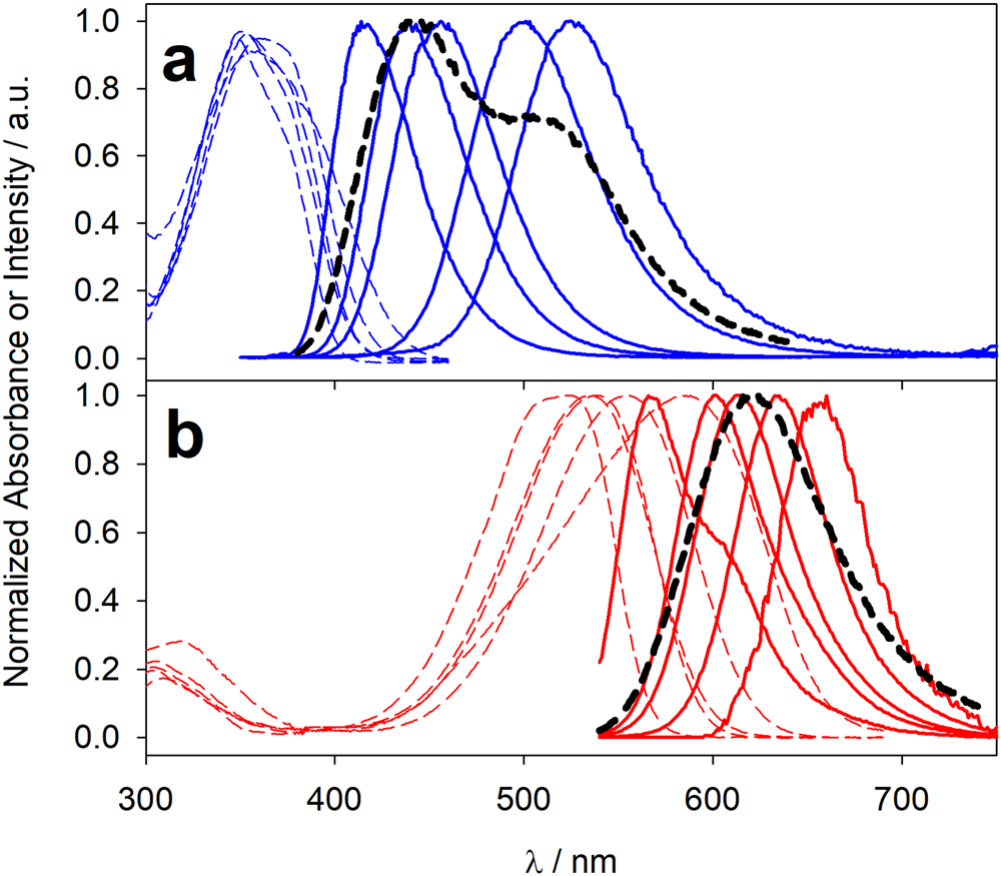
Absorption (dashed lines) and emission spectra (solid lines) of **P** (**a**, blue lines) and **NR** (**b**, red lines) in toluene, dichloromethane, acetonitrile, methanol and water (bands shifting from left to right respectively). Black dashed lines represent the emission spectra of **P** in PluS NPs (**a**), [**P**] = 5 μM, PluS NPs 1 μM) and NR in PluS NPs (**b**), [**NR**] = 2.5 μM, PluS NPs 1 μM). All spectra are normalized. Note that measured intensity of Nile Red emission in water is extremely weak compared to other solvents (see quantum yields in Table 2, not-normalized spectra *S*_*t*_*(λ)*, *S*_*d*_*(λ)*, *S*_*a*_*(λ)*, *S*_*m*_*(λ)*, and *S*_*w*_*(λ)* are shown in Fig. SI2).

We selected two well-known solvatochromic probes, i.e. Nile Red (**NR**) and Prodan (**P**) (Fig. 1), owing to their respective polarity and spectral features. They share a distinct preference for hydrophobic environments, even though they are partly soluble and emissive in polar solvents, with **P** in particular providing an appreciable signal also in water (see Table 1). Furthermore, their emission spectra show almost no overlap, even accounting for the whole range of polarities that can be explored by each probe (see normalized spectra in Fig. 2). This very low crosstalk allows for sensitive quantification of all polarity-dependent signals. Finally, Forster Radius R_0_ for **P-NR** couple is rather large for all combinations of environments, because spectral overlap increases in polar solvents, where quantum yield of the donor decreases (see Table 2). The large R_0_, in the same size range of the nanoenvironment to be probed, allows to trace back the distance between different compartments via accurate estimation of FRET efficiency between dyes. The probes were added to the dispersion of NPs via addition of small aliquots of a concentrated acetonitrile solution (1 mM), which did not interfere with the nanoarchitecture.

**Table 1:** Photophysical data for the probes **P** and **NR** in the five solvents used in this study, i.e. toluene (*t*), dichloromethane (*d*), acetonitrile (*a*), methanol (*m*) and water (*w*).

		QY%^a^	*τ* ^b^	*λ*_*em,max*_^c^	*λ*_*abs,max*_^d^	*ε* ^e^
PRODAN	*t*	56	2.21	414	348	18400
	*d*	98	3.20	438	353	24600
	*a*	95	3.30	456	350	21400
	*m*	77	2.57	501	360	19500
	*w*	25	0.98	524	359	13200
NILE RED	*t*	80	3.97	566	535	29500
	*d*	78	4.52	600	538	35500
	*a*	76	4.63	613	556	38600
	*m*	40	2.76	633	526	32800
	*w*	0,5	0.55	660	588	14400

^a^Fluorescence Quantum Yield. ^b^Fluorescence lifetime (ns), ^c^Maximum Emission Wavelength (nm), ^d^Maximum Absorption Wavelength (nm), ^e^Molar Extinction Coefficient (M^−1^ cm^−1^).

**Table 2:** Forster Radii (Å) for the FRET pair **P**-**NR** in the investigated solvent combinations.

R0		PRODAN				
		t	d	a	m	w
NILE RED	t	29.7	40.7	43.6	46.4	37.2
	d	29.1	40.0	43.3	47.9	39.7
	a	29.1	40.1	43.5	48.3	40.1
	m	26.7	37.1	40.6	46.5	39.5
	w	24.5	33.4	36.0	40.8	35.0

It is important to note that neither of the two probes display aggregation in the concentration range used in this study. Indeed, differently from known aggregates of **NR** and **P**, the emission lifetime does not feature any short component which increases with probe concentration, as it would be expected in case of aggregation. Moreover, the emission anisotropy remains high in the whole concentration range, indicating that excitation energy is not exchanged between close dyes (Figs SI1 and SI6). The rather constant spectral anisotropy (see **NR** in Fig. SI6, that contrarily to **P** does not include portions of dye in free water) also suggests that local viscosity does not change dramatically across the compartments of PluS NPs, indicating that solvent relaxation has – if any – only minor relevance in the present study. Therefore, reported spectral observations are unequivocally ascribed to change of polarity and proticity of the nanoenvironment.

### A quantitative model for heterogeneous polarity

Quantification of number and polarity of probed hosting sites is a key step of this work and a main achievement of the method presented in this contribution. First, we measured the emission spectra *S*_*i*_(*λ*) of **NR** and **P** at a fixed concentration in a set of 5 reference environments i (the pure solvents toluene, dichloromethane, acetonitrile, methanol and water, indicated as t, d, a, m, and w, respectively), covering a broad range of polarity and proticity. We choose to base our approach on the experimental emission spectra of the probes, and not on simulated spectra, in order to use information both on spectral shape and intensity (proportional to absorbance - at excitation wavelength - and quantum yield, in the specific solvent) with precision only limited by the spectrofluorometer. This allows to directly correlating the information to a physically existing environment and it is easily reproducible in different instrumental setups and conditions, while the wide selection of available solvents ensures that the best set of reference environments can virtually always be identified. The concept of “best set of reference environments” is relevant in the present method: starting from the experimental emission spectrum of the probe in the nanostructure, the reference environments must present the suitable polarities to cover the full emission spectrum and should also allow – via a simple linear combination – to obtain a good fitting of the experimental emission spectrum. This can be achieved if the polarity range is wide enough, and if the number of solvents is the minimum one that allows obtaining fitting with low residuals, i.e. falling in the condition of not underfitting nor overfitting the experimental spectrum. We selected the five solvents that allow for minimization of the residuals below a threshold of 5% (details in SI). Then, we used such reference spectra *S*_*i*_(*λ*) to fit the emission spectra of **NR** and **P** in the multicompartment nanostructure *PluSNP*(*λ*): this yields the coefficients *c*_*i*_ that quantify the fraction of dye probing the polarity and proticity of the *i*^*th*^ environment (Eq. 1).

1 $$PluSNP(\lambda )=\sum _{i=1}^{n}{c}_{i}{S}_{i}(\lambda )$$

### Single-probe polarity study

**Figure 3:**
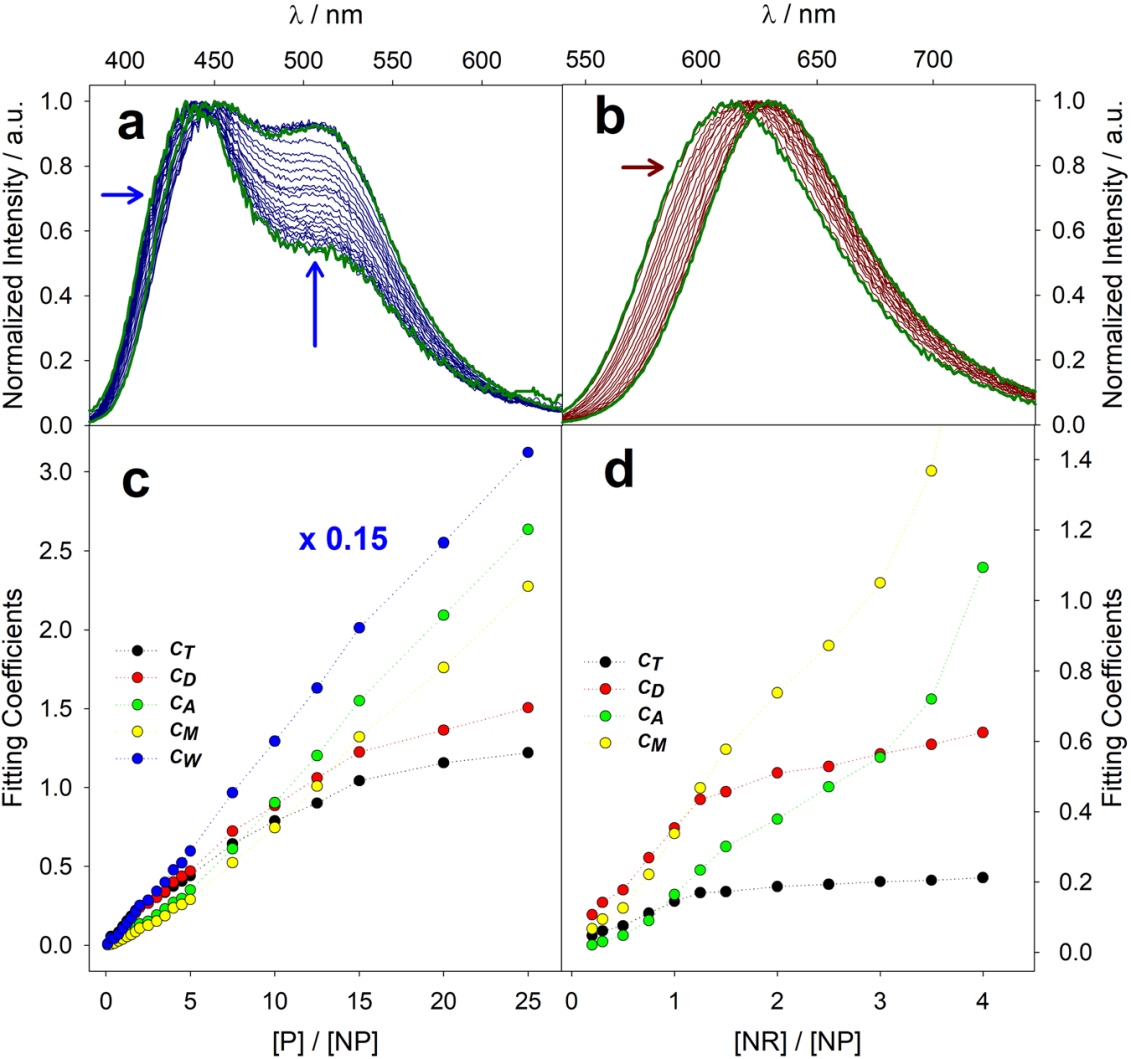
Normalized emission spectra of titration of Plus NPs (1 μM) with P (**a**, 0.2–25 μM) and NR (**b**, 0.2–4 μM) and fitting coefficients of the spectral components relative to toluene (*c*_*t*_, black), dichloromethane (*c*_*d*_, red), acetonitrile (*c*_*a*_, green), methanol (*c*_*m*_, yellow) and water (*c*_*w*_, blue), obtained from the not-normalized emission spectra of titration of Plus NPs with P (**c**) and NR (**d**). First and last titration spectra are shown in green for clarity. *c*_*w*_ is shown multiplied by a coefficient 0.15 to facilitate data visualization.

Both **NR** and **P**, when added in small amounts to a water solution of Plus NPs (1·10^−6^M), can be housed in a heterogeneous variety of hosting sites and therefore generate complex (multicomponent) signals. The information obtained by the two probes is complementary since they exhibit different spectral responses and cover different ranges of polarity. The gradual shift of the emission maximum displayed by **NR** upon increasing its concentration in the presence of PluS NPs reveals that polarity of populated hosting sites shifts progressively from an environment similar to toluene to one comparable to methanol (see normalized emission spectra in Fig. 3b and not normalized spectra in Fig. S3). On the other hand, **P** displays a peculiar emission spectrum with two well separated bands, which respectively report on aprotic (higher energy emission band) and protic microenvironments (lower energy emission band)^18,19^. Interestingly, each band is also sensitive to polarity, so that it is possible to distinguish among strongly and mildly apolar environments (corresponding to toluene and dichloromethane in our set of 5 reference solvents) and among mildly and strongly hydrophilic sites, up to a purely aqueous environment (acetonitrile, methanol and water, see Fig. 3a). Differently from **NR**, **P** displays, even at the lowest concentration, a significant contribution of polar protic environments (lower energy band), whose weight increases with the increase of **P** concentration with a concomitant relevant bathochromic shift (Fig. 3a). Coefficients found upon fitting the experimental emission spectra of **P** and **NR** show comparable trends: coefficients of toluene and dichloromethane (*c*_*t*_ and *c*_*d*_) show an initially high slope (indicating efficient population of hydrophobic hosting sites) which decreases, until reaching a plateau, at high probe concentration, indicating the saturation of such hydrophobic sites even at relatively low probe amounts. On the other hand, the more hydrophilic hosting sites, which are here monitored via the coefficients of acetonitrile and methanol (*c*_*a*_ and *c*_*m*_ respectively) are able to host a larger number of dyes: the coefficients *c*_*a*_ and *c*_*m*_ overcome *c*_*t*_ and *c*_*d*_ respectively, and do not reach a plateau at least up to 25 μM. This analysis thus shows that the two solvatochromic probes **P** and **NR** are, at low concentrations, hosted by both hydrophilic and hydrophobic sites of PluS NPs, with a preference for hydrophobic sites; then, at higher probe concentration, hydrophobic sites are saturated, and the probes largely populate hosting sites that are as hydrophilic and protic as a methanol environment. **P** also features a rather large coefficient for water (*c*_*w*_, Fig. 3) since the very first additions, which deserves separate considerations.

**P** is indeed clearly reporting on a water-like microenvironment: this might correspond to either a water pocket inside the PEG shell, or simply to a partial solution and persistence of **P** in the bulk water outside PluS NPs.It is very important to shine light on this point since the presence of water molecules inside the PEG shell is crucial for many applications of PluS NPs and of PEG stabilized nanostructures in general: they can for instance be involved in catalytic reactions taking place in the nanocompartment, they can stabilize or decompose hosted drugs, they can regulate kinetics of delivery or transport and permeation of small molecules and ions inside nanostructures^37,38^.

### “FRET probe”: nanopolarity mapping

To unambiguously localize the hydrated compartments with respect to the hydrophobic ones, we took advantage from the FRET processes between the two solvatochromic probes. This approach allowed us to determine whether the polar protic environments (here quantitatively described by *c*_*m*_ and *c*_*w*_) are reporting on hydrated molecules inside PEG shell or on bulk water outside the PluS NPs. We loaded 5·10^−6^ M **P** in Plus NPs, corresponding to an average of 5 **P** dyes per NP, so that the environments probed by the donor **P** dyes in these conditions could cover all polarities, including water. Then, we added increasing amounts of **NR** to this system, following its localization via its selective excitation at 520 nm: the emission titration (Fig. SI4) was very similar to the one obtained for **NR** alone (Fig. 3b), suggesting that the presence of small amounts of **P** did not interfere with distribution of **NR**. On the other hand, upon direct excitation of **P** at 330 nm, we observed a pronounced quenching of **P** emission and a concomitant sensitization of **NR**. This preliminary observation confirms that the two probes are located within the same nanostructure and thus close enough to allow an efficient energy transfer process, also owing to their rather large R_0_ (Table 2).

**Figure 4:**
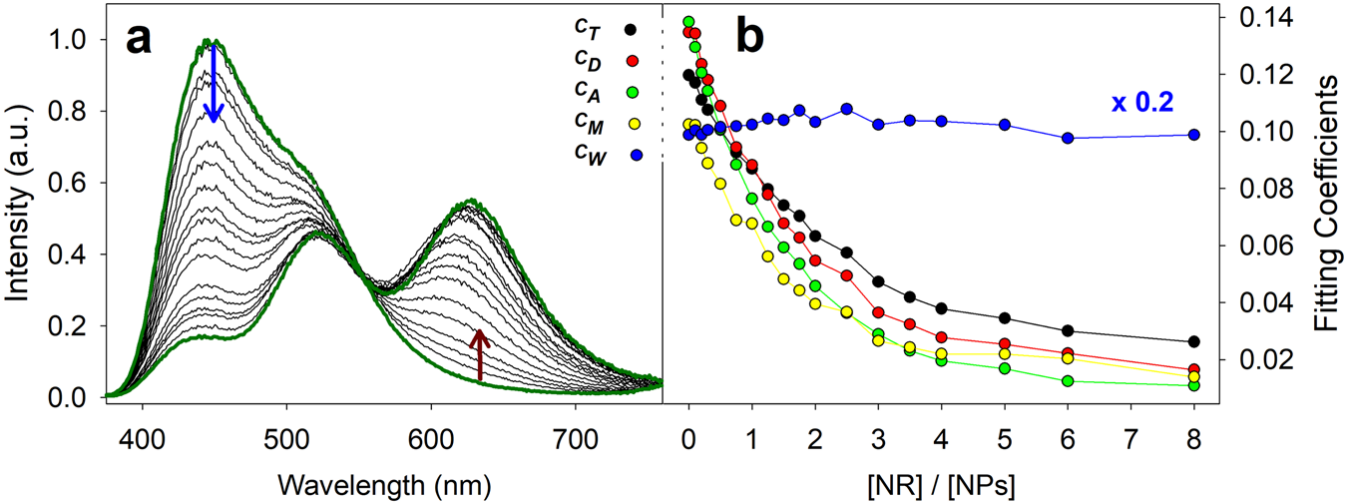
Normalized emission spectra (**a**) and fitting emission coefficients (**b**) of FRET titration of PluS NPs (1 μM) and P (5 μM) with increasing amounts of NR (0.1–12 μM). First and last titration spectra are shown in green for clarity.

Beside this general observation, our spectral deconvolution method allowed us to resolve the various polarities probed by **P** and **NR**, and consequently to evaluate FRET efficiency as a function of the microenvironment. This procedure opens the way to a fine understanding of the system, since it reveals whether **P** is transferring energy to **NR** from all probed compartments, including the hydrated (protic) ones. We analyzed the quenching of **P** after separating its complex emission spectrum in the 5 components and then we follow the trend of the coefficients, with particular attention to *c*_*m*_ and *c*_*w*_. Interestingly, while the coefficients *c*_*t*_, *c*_*d*_, *c*_*a*_ and *c*_*m*_ are heavily depleted by the increasing concentration of **NR**, *c*_*w*_ remains substantially constant (Fig. 4b).

This analysis led us to draw two important conclusions. First, nanoenvironments spanning a wide range of polarity and proticity are very close to each other (toluene-, dichloromethane-, acetonitrile- and methanol-like): the high FRET efficiency (the various components of **P** emission are quenched by approximately 90% by only 3-5 acceptor molecules per nanoparticle) prove that the relative distance between compartments is shorter than R_0_ (which ranges from 3 to 5 nm, see Table 2). In particular, this method demonstrates the presence of a compartment of PluS NPs featuring polar protic hosting sites (methanol-like nanoenvironments), which can be ascribed to PEG branches hydrated by a restricted number of water molecules. On the other hand, the “water-like” compartment probed by **P** is not affected by FRET processes therefore it has to be far away from the other hydrophobic and hydrophilic sites hosting the **NR** dyes, and thus can be ascribed to bulk water, external to the nanostructure, able to partially solubilize **P**; this rules out the presence of water pockets inside the nanostructure.

In order to experimentally prove the consistency of our findings, we have performed an analogous polarity-resolved spectral analysis of FRET on the acceptor, **NR**. Deconvolution of directly excited NR spectra (λ_exc_ = 520 nm) and of sensitized **NR** spectra (i.e., excited via FRET from **P**, λ_exc_ = 330 nm) provides coefficients of environments probed by **NR** and coefficients of the environments in which **NR** accepts energy from **P**, respectively. Quantitative comparison of such coefficients reveals that all components of **NR** are equally sensitized by **P**, confirming that all emissive **NR** probes are distributed inside the different microenvironments of PluS NPs – from apolar (*c*_*t*_ and *c*_*d*_) to polar (*c*_*a*_) and polar protic ones (*c*_*m*_) – (Fig. SI4). In addition, emission anisotropy measurements support these findings: anisotropy of **P** drops close to zero in the highest wavelength region of the spectrum, indicating that the probes emitting in that region have much higher rotational diffusion than the others (Fig. SI5). This is instead not observed for NR, which displays a constantly high emission anisotropy (Fig. SI6).

The highly informative results collected with this method demonstrate that the two-compartment model (silica core/PEG shell) used so far to describe the architecture of Plus NPs is highly oversimplified. In fact, the hosted molecules were able to probe and localize a variety of nanoenvironments within each nanostructure, featuring very different polarity and proticity. A more rigorous description of the system should hence consider a thin apolar region (matching with the inner layer of dense, not hydrated PPO), and an increasingly polar layer (ascribable to PEG branches) with a volume able to host tens of dyes. The polar layer reaches, in the outer part, a very high polarity and proticity, comparable to the ones of methanol: this evidence allows us to ascribe this nanoenvironment to strongly hydrated PEG branches.

## Conclusions

In conclusion, we developed a new method to investigate the multifaceted polarity and proticity of complex nanostructures, with the unique ability of co-localizing the different probed environments even in such a confined space. The method is based on the use of at least two solvatochromic fluorescent probes with suitable photophysical properties (including R_0_ in the same size range as the distance between the compartments to be probed) to efficiently exchange energy via FRET. These species can hence (i) report on the presence of multiple environments within the nanoarchitectures, and (ii) colocalize the various environments via FRET. The method has been tested here on a well-known multicompartment nanomaterial – PluS NPs – using two solvatochromic probes, Prodan and Nile Red. We found a wide range of polarities, ranging from apolar, to polar, and also to polar protic nanoenvironments, within R_0_ distance (3–5 nm in the present study), ascribable to dense PPO, PEG and to partly hydrated PEG branches respectively. In addition, it was possible to experimentally prove that the water-like component detected by **P** does not belong to the nanostructure, but it is merely due to bulk water.

This simple method is general and can be applied in a versatile, modular fashion to a broad range of nanomaterials, but it should be taken into account that the spectral response can partially depend not only on polarity but also on differences in solvent relaxation across the compartments of nanomaterials^39^. We believe that it could be of inspiration for a wide scientific community involved in fabricating, investigating and using nanostructured materials, since understanding polarity distribution in these systems is essential to grasp their potential in a variety of fields and applications, ranging from their use as nanoreactors and artificial enzymes, to molecular adsorption, storage and release as required in drug delivery applications.

## Methods

### Chemicals

All reagents and solvents were used as received without further purification: non-ionic triblock surfactant Pluronic PF127, tetraethylortosilicate (TEOS, 99.99%), chlorotrimethylsilane (TMSCl, ≥98%), Acetic acid (≥99.7%), Prodan **P** (N,N-Dimethyl-6-propionyl-2-naphthylamine, ≥98.0%) and Nile Red **NR** (≥98.0%) were purchased from Aldrich. Reagent grade NaCl was purchased from Fluka. A MilliQ Millipore system was used for the purification of water (resistivity <18 MΩ).

### Synthesis of PluS NPs

Plus NPs have been synthesized as previously reported^31–36,40^. Briefly, tetraethylorthosilicate (TEOS, 180 µL, 0.8 mmol) was added to an acidic aqueous solution (Acetic Acid 1 M) of Pluronic F127 (100 mg) and NaCl (67 mg) under magnetic stirring, and its hydrolysis and condensation was allowed to proceed for 3 hours. Trimethylsilylcloride (TMSCL, 10 µL, 0.08 mmol) was then added and the solution was stirred overnight. The resulting Plus NPs dispersion was purified via dialysis for at least 4 days, using RC membrane (cut off 12 KDa) against water. The dispersion in the membrane was recovered and diluted to 10 ml, yielding a final PluSNP concentration of 10 µM^34^. The morphology of PluS NPs was characterized with DLS and TEM, yielding the hydrodynamic radius (R_H_ = 13 nm, polydispersity index PdI = 0.11) and the diameter of the silica core (d_c_ = 11 ± 1 nm), respectively. Representative DLS results and TEM image used for image analysis are shown in Fig. 1 and in Fig. SI1.

### Fluorescence titrations of PluS NPs with solvatochromic probes

2.5 mL of 1·10^−6^ M solution of PluS NPs in water was titrated with a small amount (1 µL to 50 µL) of a millimolar acetonitrile solution of solvatochromic dye (**P** or **NR**). Absorption spectra were recorded on a Perkin-Elmer Lambda 45 spectrophotometer, while emission spectra were recorded on an Edinburgh F900 spectrofluorometer equipped with a photomultiplier Hamamatsu R928P. FRET experiments were performed by titrating an aqueous solution of PluS NPs 1·10^−6^ M and **P** 5·10^−6^ M with a millimolar solution of **NR** in acetonitrile.

### Data analysis

Spectral deconvolution was performed through the fitting algorithm of Sigmaplot (Systat Software Inc.) following Eq. 1 (more info in SI). Combinations of spectra of **P** and **NR** in different solvents (experimentally obtained respectively in toluene, dichloromethane, acetonitrile, methanol and water) were used to fit the spectra of **P** and **NR** in PluS NPs. The solvents were chosen for their distribution in polarity, and found to be a promising set for high quality fitting of spectra of **P** and **NR** in PluS NPs. Details on the fitting tests for selection of solvents set can be found in SI. Experimental input spectra *S*_*i*_*(λ)* were recorded at equimolar concentration of solvatochromic probe in five selected solvents, using constant instrumental conditions and excitation wavelengths (λ_exc_ = 330 nm for **P** and λ_exc_ = 520 nm for **NR**). The water component was not used to fit **NR** emission spectra, due to the very low quantum yield and solubility of this solvatochromic probe in this solvent.

## Electronic supplementary material

Supplementary information
