# Supplementary material for: Mapping heterogeneous polarity in multicompartment nanoparticles
Source: Sci Rep. 2018 Nov 20;8:17095. doi: 10.1038/s41598-018-35257-y (PMC6244083; doi:10.1038/s41598-018-35257-y)
Supplement: Supplementary file 1 — Supplementary information [file 41598_2018_35257_MOESM1_ESM.docx]

**Supporting Information**

Mapping heterogeneous polarity in multicompartment nanoparticles

Francesco Palomba, Damiano Genovese, Luca Petrizza, Enrico Rampazzo, Nelsi Zaccheroni and Luca Prodi

Dipartimento di Chimica “G. Ciamician”, Università di Bologna, via Selmi 2, 40126, Bologna, Italy

**INDEX**

- *TEM micrographs*
- *Fluorescence spectra of* ***NR*** *and* ***P*** *in solvents and PluS NPs*
- *Fluorescence anisotropy measurements*
- *Data analysis*
- *Selection of the set of reference solvents*


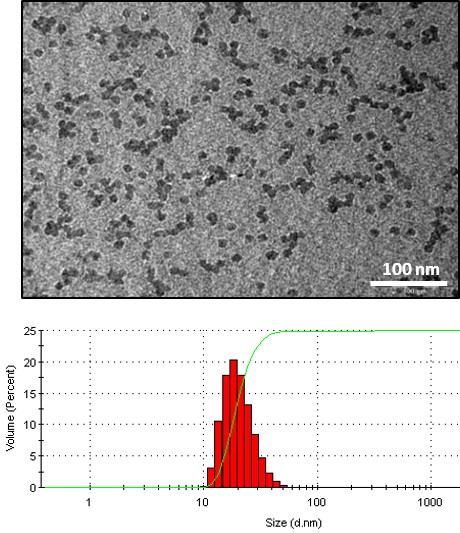


Figure SI1 – TEM micrograph of PluS NPs.


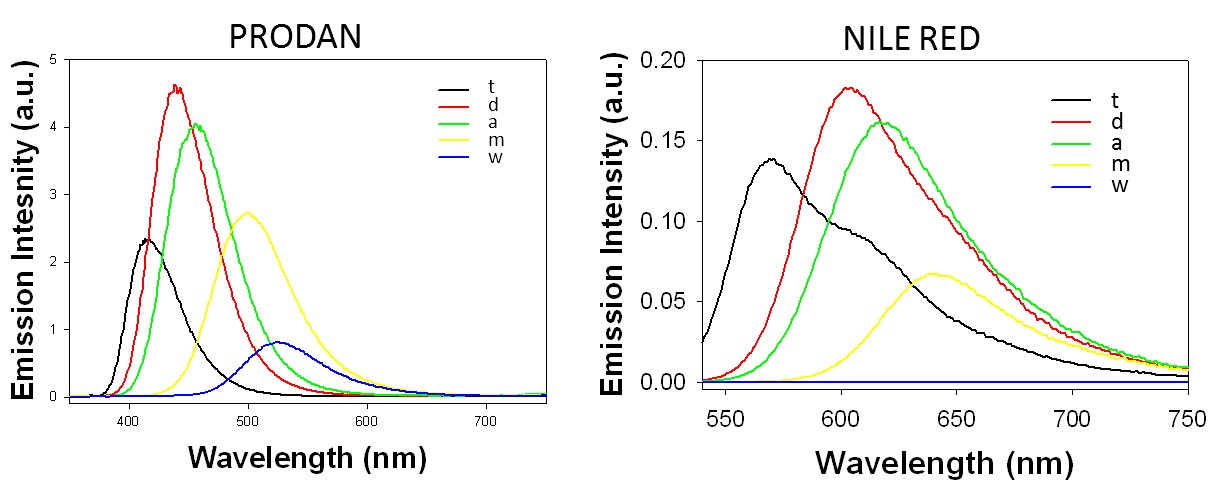
Figure SI2 – Emission spectra of equimolar solutions of Prodan (left) and Nile Red (right) in the five solvents used in this study (t=toluene, d=dichloromethane, a=acetonitrile, m=methanol, w=water). Excitation wavelength is 330 nm and 520 nm for Prodan and Nile Red respectively. Concentration of the probes is 5 μM.


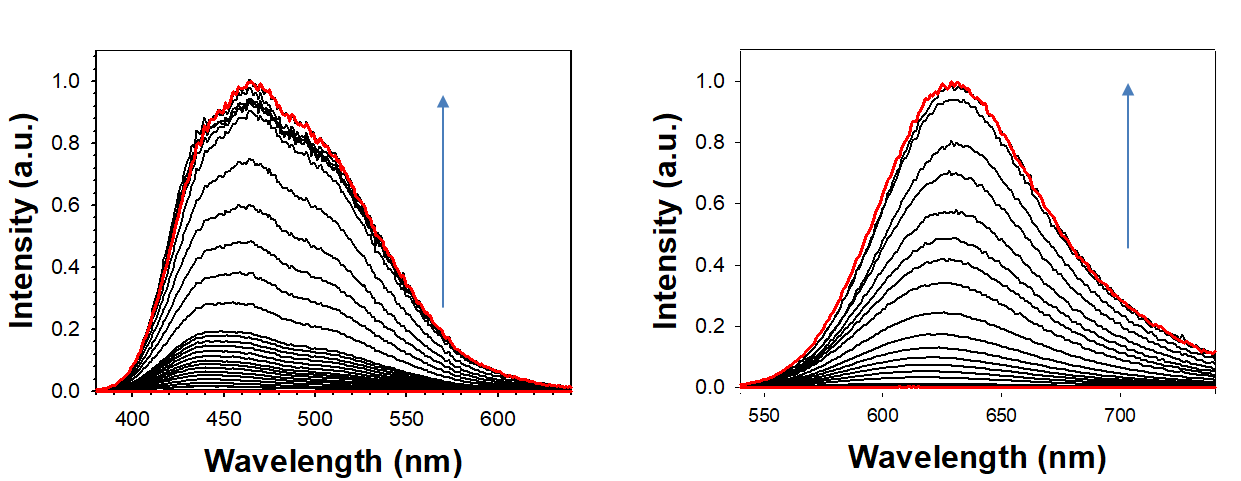


Figure SI3 – Emission spectra (not normalized) of PluS NPs 10^-6^ M in water titrated with Prodan (excitation wavelength 330 nm, 0.2 – 25 μM) or with Nile Red (excitation wavelength 520 nm, 0.2 - 4 μM, right).


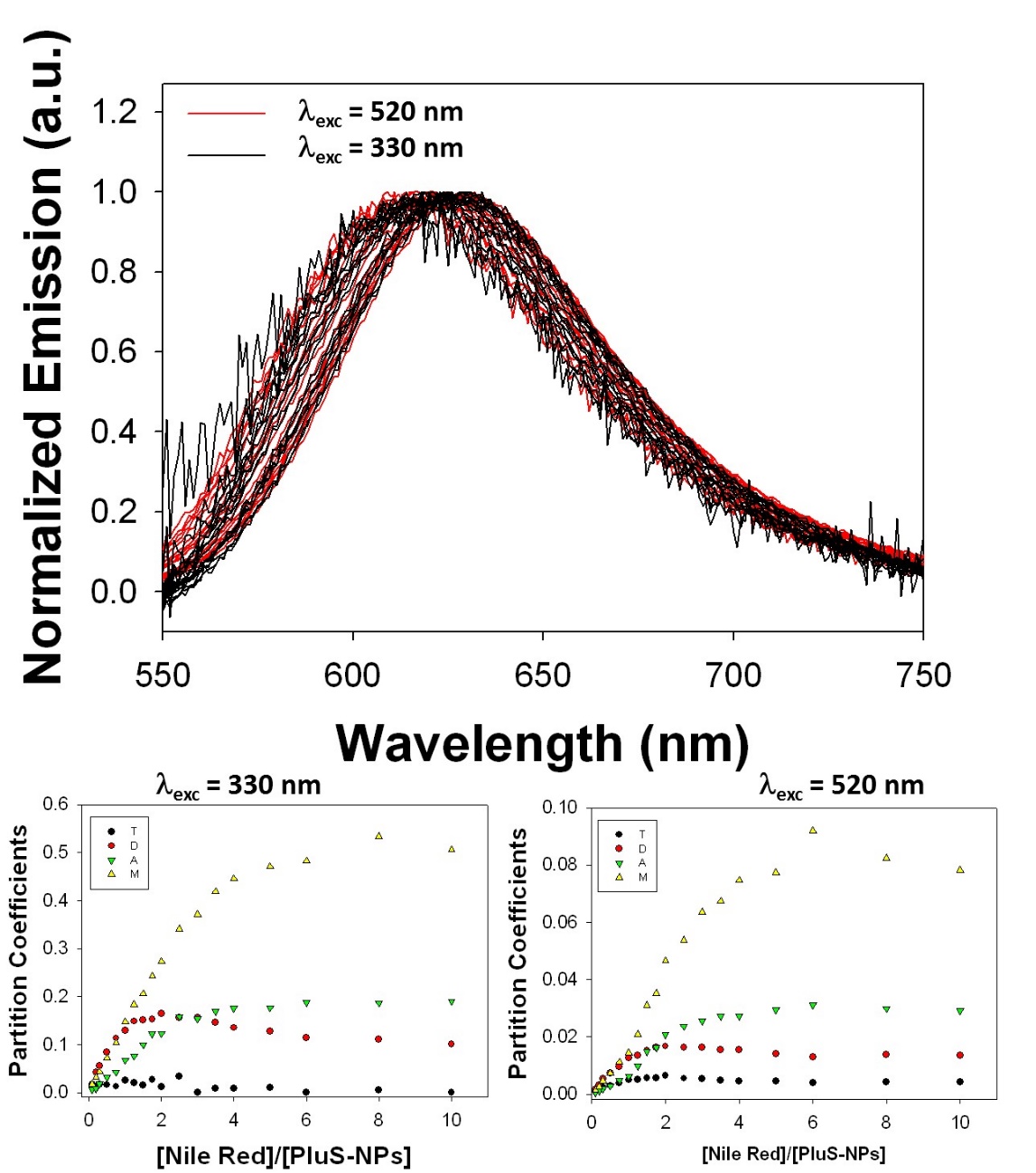


Figure SI4 – Top graph: normalized emission spectra of Nile Red (0.1 - 12 μM) added to a solution containing PluS NPs 10^-6^ M and Prodan 5*10^-6^ M, excited directly at 520 nm (red spectra) or excited via FRET through Prodan at 330 nm (black spectra). The latter spectra were obtained by measuring the overall emission spectra which includes emission from Prodan (shown in figure 4), followed by fitting the spectra with the nine spectral components (5 for Prodan and 4 for Nile Red), and finally subtracting the emission components relative to Prodan to the original spectra shown in figure 4, hence yielding uniquely the sensitized Nile Red emission. Bottom graphs: fitting coefficients ct, cd, ca and cm obtained for emission spectra reported above, i.e. for sensitized (left) and for directly excited Nile Red (right).

***Fluorescence anisotropy measurements***

All fluorescence anisotropy measurements were performed on an Edinburgh FLS920 equipped with Glan-Thompson polarizers. Anisotropy measurements were collected using an L-format configuration, and all data were corrected for polarization bias using the G-factor.

Four different spectra were acquired for each sample combining different orientation of the excitation and emission polarizers: *I*_VV_, *I*_VH_, *I*_HH_, *I*_HV_ (where V stands for vertical and H for horizontal; the first subscript refers to the excitation and the second subscript refers to the emission). The spectra were used to calculate the G-factor and the anisotropy *r*:

*G* = *I*_HV_/*I*_HH_

*r* = (*I*_VV_ - *GI*_VH_)/*I*_VV_ + 2*GI*_VH_


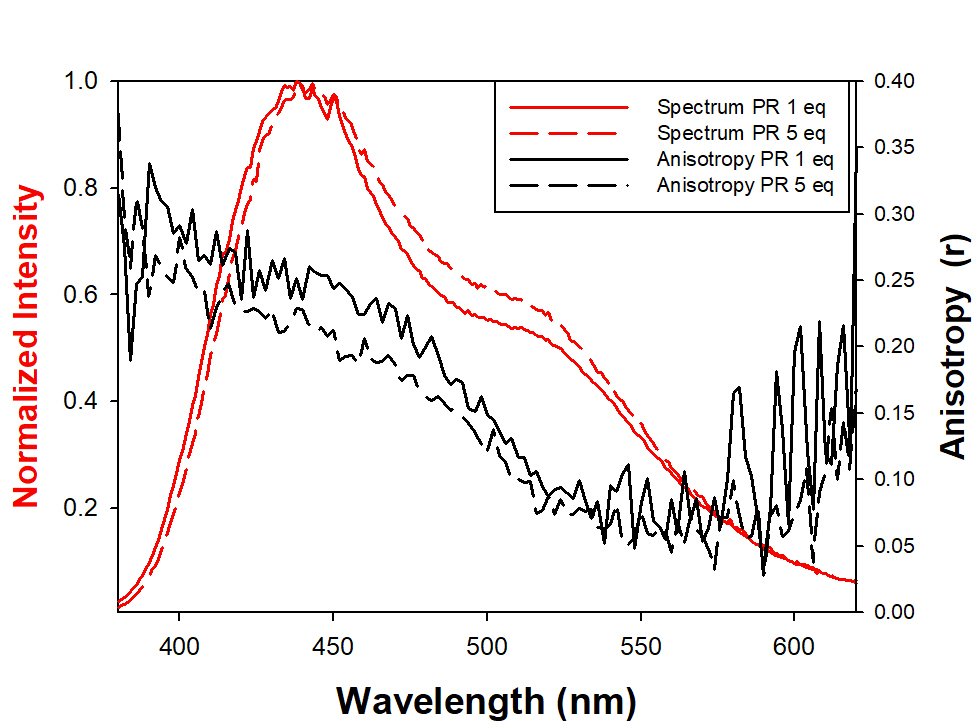


Figure SI5 - Fluorescence Anisotropy (black line) and Normalized Fluorescence Emission intensity (red line) of of Prodan 1 and 5*10^-6^ M (solid and dashed respectively) added to 1*10^-6^ M of PluSNPs (λ_exc_ = 330 nm).


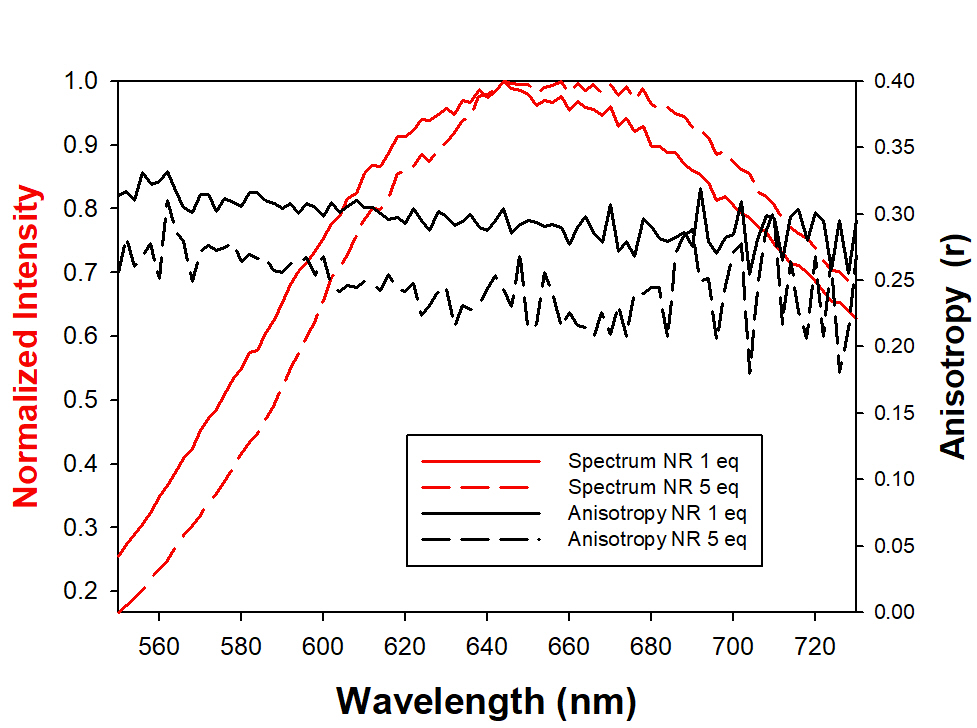


Figure SI6 - Fluorescence Anisotropy (black line) and Normalized Fluorescence Emission intensity (red line) of of Nile Red 1 and 5*10^-6^ M (solid and dashed respectively) added to 1*10^-6^ M of PluSNPs (λ_exc_ = 520 nm).

***FRET analysis***

Forster Radii are calculated for all combinations of donor and acceptor couples, where the donor dye is Prodan in the five solvents (toluene, dichloromethane, acetonitrile, methanol and water) and the acceptor dye is Nile Red in the same five solvents. To do so, a known small volume (5 microliters) of a concentrated methanol solution of the dye is dissolved in a cuvette containing a large volume of a solvent (2.5 mL), in order to obtain diluted solutions of the dyes in the five solvents. The molar extinction coefficient spectra, the emission spectra and the quantum yields were measured for each sample and used for calculation of the Forster Radius using the following equation,

$$R_{0}=\sqrt[6]{Q_{D}\kappa^{2}\left( \frac{9000ln10}{128 \pi^{5}Nn^{4}} \right)J\left( \lambda\right)}$$

where Q_D_ is the quantum yield of the donor dye, $\kappa$ ^2^ the orientation factor, N the Avogadro number, n the refractive index and J the overlap integral:

$J(\lambda)=\int_{0}^{\infty} F_{D}\left( \lambda\right)\varepsilon_{A}\left( \lambda\right)\lambda^{4}d\lambda$

where *F_D_(*$\lambda$*)* and $\varepsilon_{A}\left( \lambda\right)\lambda^{4}$*)* are the normalized fluorescence intensity and molar extinction coefficient spectra respectively, and $\lambda$ the wavelength. [Lakowicz, J. R. *Principles of Fluorescence Spectroscopy*; Kluwer Academic/Plenum Publishers: New York, 1999] In the system under study we assumed, for all combinations of donor and acceptor dyes, that the orientation factor of immobilized and randomly oriented dyes applies, yielding an average $\kappa$ ^2^ of 0.476. This assumption is supported by the high fluorescence anisotropy (0.2 < *r* < 0.3) measured for both Prodan and Nile Red in the concentration range used in this study.

***Data analysis***

Spectral deconvolution was performed through the fitting algorithm of Sigmaplot (Systat Software Inc.) following eq. 1. The SigmaPlot curve fitter uses the Marquardt-Levenberg algorithm to find the coefficients (parameters) of the independent variable(S) that give the best fit between the equation and the data. This algorithm iteratively seeks the values of the parameters that minimize the sum of the squared differences between the values of the observed and predicted values of the dependent variable.

Input spectra *S_i_(λ)* were recorded at equimolar concentration of solvatochromic probe in the five selected solvents, using constant instrumental conditions and excitation wavelengths (λ_exc_ = 330 nm for **P** and λ_exc_ = 520 nm for **NR**). The water component is not used to fit **NR** emission spectra, due to the very low quantum yield and solubility of this solvatochromic probe in this solvent.

***Selection of the set of reference solvents***

The emission spectra measured with 5 equivalents of **P** and **NR** respect to PluS NPs were fitted with combinations of the five reference spectra of **P** and **NR** respectively in toluene, dichloromethane, acetonitrile, methanol and water. Combinations of 1, 2, 3, 4 and 5 spectra were used. The residuals were integrated vs wavelength and divided by the spectrum integral, so to obtain the % fitting residuals, which are plotted in figure SI7. The % residuals decrease down to less than 1%, a value that we arbitrary choose as the threshold, when the number of reference spectra increases from 1 to 5. The experimental and fitted spectra, together with residuals, are shown in figures SI8 and SI9, which clearly demonstrate that 5 solvents, well distributed in polarity, give the minimum number of reference spectra to obtain fitting of complex polarity spectra with low residuals.

Figure SI7. Trends of Residuals Integral% (Residuals integral/ Experimental Spectrum Integral *100) of the fitting with different basis sets for Prodan (black line) and Nile Red (red line) 5 *10^-6^ M in presence of PluSNPs 1*10^-6^ M.


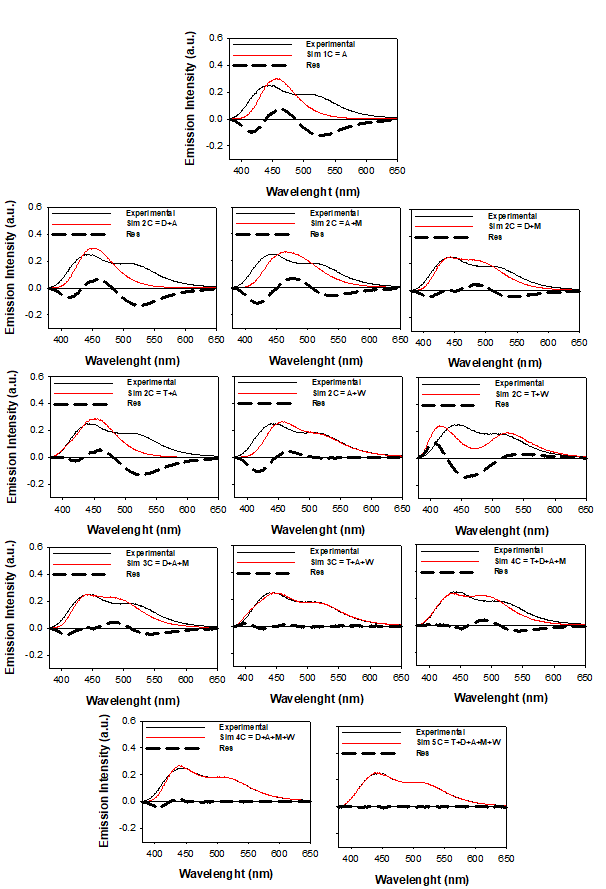


Figure SI8 - Display of the fitting combinations for Prodan 5 *10^-6^ M in presence of 1*10^-6^ M of PluSNPs. From the top to the bottom different composition of the basis set for the fitting are showed, in particular, from one component A to the five components T, D, A, M and W. The black solid line represents the experimental spectra in red the fitted one. The dashed black line rapresent the residuals (fitted minus the experimental spectra).


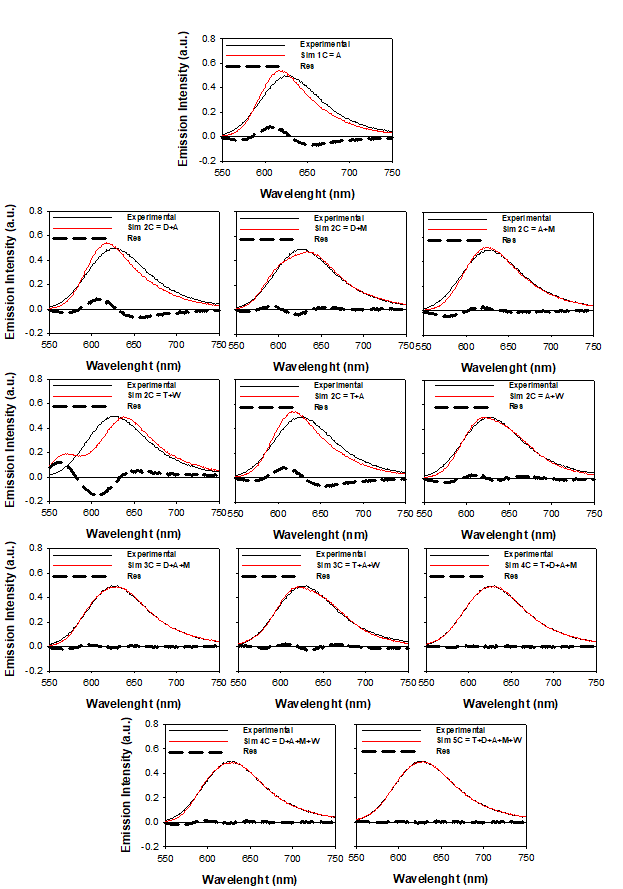


Figure SI9 - Display of the fitting combinations for Nile Red 5 *10^-6^ M in presence of 1*10^-6^ M of PluSNPs. From the top to the bottom different composition of the basis set for the fitting are showed, in particular, from one component A to the five components T, D, A, M and W. the black solid line represent the experimental spectra in red the fitted one. The dashed black line rapresent the residuals (fitted minus the experimental spectra).
